# Innate sensitivity to face-to-face biological motion

**DOI:** 10.1016/j.isci.2024.108793

**Published:** 2024-01-04

**Authors:** Mirko Zanon, Bastien S. Lemaire, Liuba Papeo, Giorgio Vallortigara

**Affiliations:** 1 Center for Mind/Brain Sciences, University of Trento, Rovereto, Italy; 2 Institut des Sciences Cognitives - Marc Jeannerod, UMR5229, Centre National de la Recherche Scientifique (CNRS) & Université Claude Bernard Lyon 1, France

**Keywords:** Neuroscience, Developmental neuroscience, Social sciences

## Abstract

Sensitivity to face-to-face stimuli configurations, which likely indicates interaction, seems to appear early in infants’ development, and recently a preference for face-to-face (vs. other spatial configurations) has been shown to occur in macaque monkeys. It is unknown, however, whether such a preference is acquired through experience or as an evolutionary-given biological predisposition. Here, we exploited a precocial social animal, the domestic chick, as a model system to address this question. Visually naive chicks were tested for their spontaneous preferences for face-to-face vs. back-to-back hen dyads of point-light displays depicting biological motion. We found that female chicks have a spontaneous preference for the facing interactive configuration. Males showed no preference, as expected due to the well-known low social motivation of males in this highly polygynous species. These findings support the idea of an innate and sex-dependent predisposition toward social and interacting stimuli in a vertebrate brain such as that of chicks.

## Highlights

• Naive female chicks approach face-to-face rather than back-to-back point-light hens
• It reveals a biological predisposition to recognize socially relevant configurations
• Modulation by sex could be driven by the polygynous mating system of the species

## Graphical abstract

**Figure:**
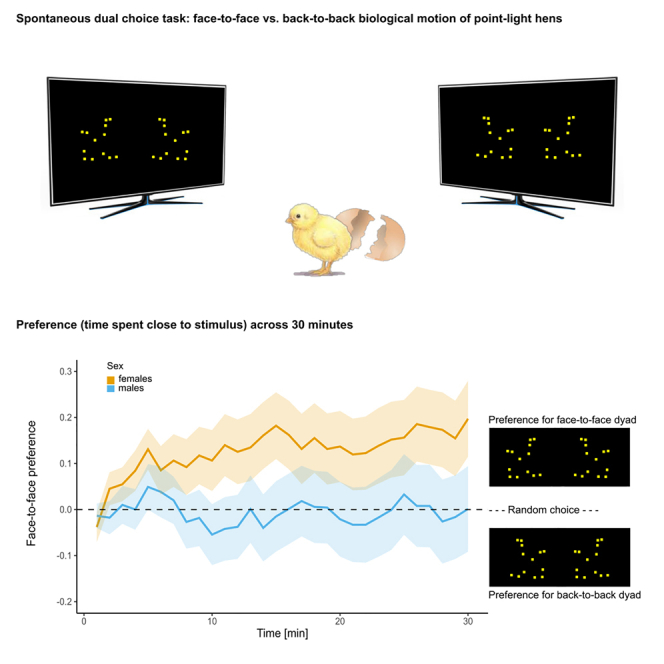

## Introduction

The brains of young vertebrate animals are equipped with animacy detectors, mechanisms to recognize the presence of other animals in the environment. For example, newborn humans and newly hatched chicks quickly detect face-like stimuli^1^^,^^2^^,^^3^ and exhibit preferences for self-propelled^4^^,^^5^^,^^6^^,^^7^^,^^8^^,^^9^ and biological motion.^10^^,^^11^^,^^12^^,^^13^^,^^14^^,^^15^

This sensitivity to animacy signals not only helps organisms to spot the presence of other living beings in their vicinity but also lays the foundation for the development of social interactions.^16^^,^^17^ Stimuli involving multiple agents that interact with each other may provide information about social events. One example is when two bodies are positioned face-to-face and therefore suggest an interaction compared to a back-to-back positioning.^18^^,^^19^

In humans, visual sensitivity to the difference between face-to-face and back-to-back bodies appears early in development^18^ and evolves into a preference for face-to-face bodies, which is shared with monkeys.^20^ In those studies, the looking times of six-month-old infants were computed while presenting simultaneously two dyads showing face-to-face and back-to-back bodies, respectively, finding a significant difference in looking times toward the two types of dyads.^18^ The same looking-time paradigm was adopted in adult humans and macaques, confirming the different attention driven by the two types of stimuli, and thus, the relevance of such configurations.^20^ Those results are compatible with the evidence of perceptual adaptation for efficient processing of seemingly interacting (e.g., face-to-face) bodies. In fact, it has been shown that, in conditions of visual noise, facing bodies are more likely to be detected and recognized than the same two bodies facing away from each other.^21^^,^^22^ Other studies showed that the face-to-face positioning of bodies impacts the very early, preattentive stages of visual perception,^19^^,^^23^ up to visual memory.^24^^,^^25^ The behavioral advantage in processing facing people has a counterpart in neuroimaging results showing that, in humans, body-selective regions of the visual cortex respond more strongly to multiple bodies that appear to be interacting (i.e., face-to-face), relative to unrelated bodies.^26^^,^^27^

However, whether the visual preference for face-to-face bodies is acquired through experience or rather represents an example of an evolutionarily given predisposition is unknown, and it is the issue we aim to address here.

**Figure 1:**
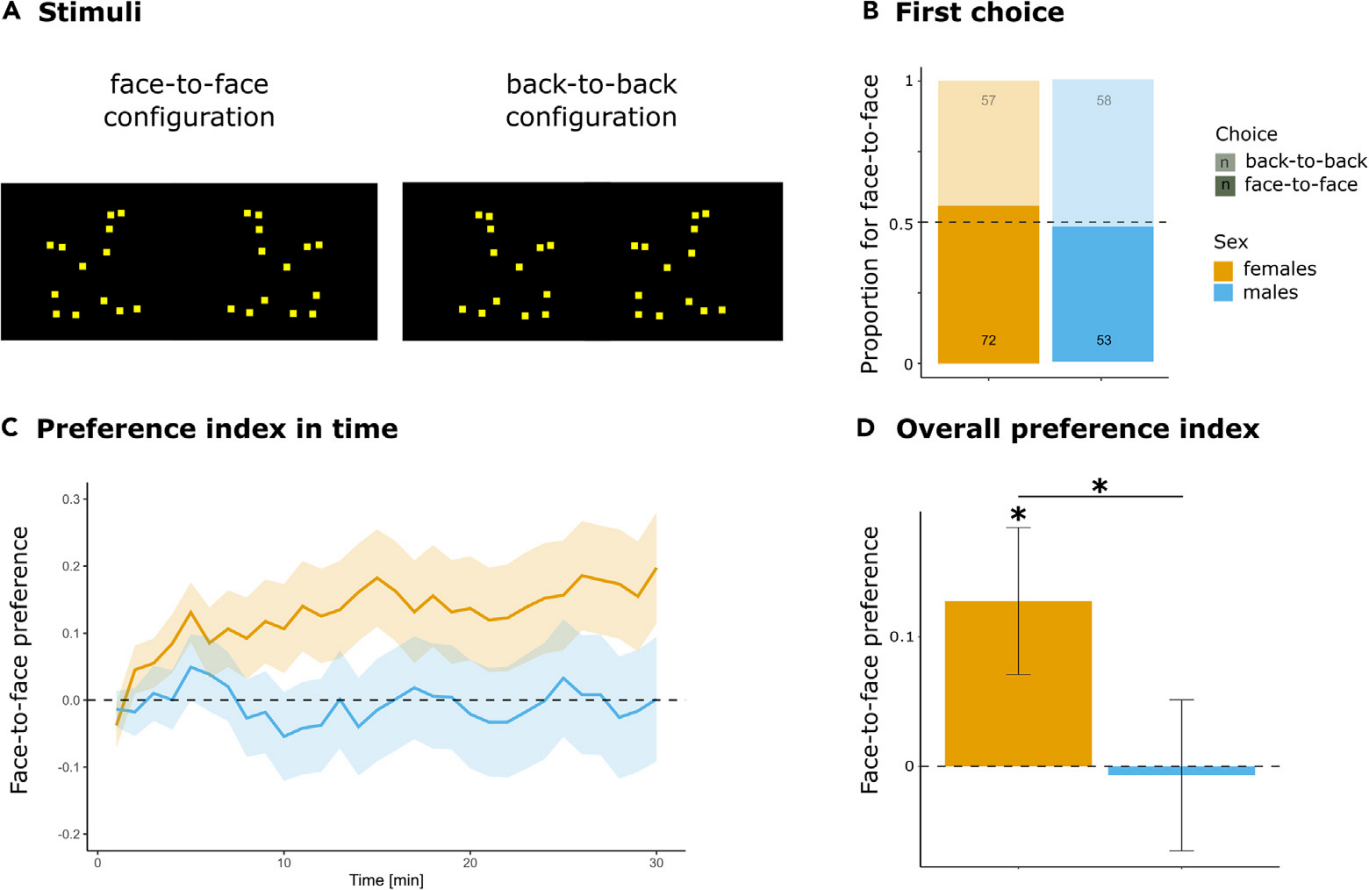
Stimuli and chicks’ spontaneous choice (A) Example of a frame for face-to-face (left) and back-to-back (right) configurations. (B) First choice proportion for face-to-face configuration. Proportion of time spent to face-to-face configuration (preference index) as a function of time (C) and overall for the 30 min of test (D; mean±se, ∗ p < 0.05). GIF files with the moving stimuli are available in the data repository Figshare: https://figshare.com/articles/dataset/Innate_sensitivity_to_face-to-face_biological_motion_dataset/23713299.

We tested newly hatched visually naive chicks for their spontaneous preferences for a pair of biological motion patterns representing two point-light hen silhouettes walking either face-to-face or back-to-back (Figure 1A). We measured the first approach and time spent near the two stimuli in both male and female newly hatched chicks.

## Results

To test whether the chicks were more likely to first approach the face-to-face over the back-to-back stimuli, we tracked the animals’ first choice (Figure 1B).

No statistically significant differences were observed in the first approach (between sexes comparison **χ**^2^(1) = 1.25, p = 0.26; overall binomial probability for facing configuration 0.52, 95% CI: 0.46–0.59, p = 0.5).

After the first approach, choice proved to stabilize over time. Indeed, we performed a longitudinal analysis with time and sex as factors, to investigate whether response changes across the 30 min of test. This did not reveal any significant main effect (time: 1.5, df = 3.6, p = 0.2; sex 2.5, df = 1, p = 0.1; interaction: 0.8, df = 3.6, p = 0.5). Still, a trend diverging from random choice after the first minutes could be observed in female (Figure 1C).

To test whether the chicks were more likely to spend most of the overall time close to the face-to-face over the back-to-back stimuli, we computed the proportion of time animals spent close to the face-to-face configuration during the overall 30 min of the experiment (Figure 1D). In addition, we considered again sex as a factor, taking into account previous evidence of sex differences in the interaction with social stimuli.^28^^,^^29^^,^^30^^,^^31^

A permutation test on the proportion of time spent close to the stimuli revealed a sex difference (permutation p = 0.047, effect size Cohen’s d = 0.2). Females spontaneously chose to spend more time near the face-to-face configuration (Z = −2.16, p = 0.03, effect size Cohen’s d = 0.2; post-hoc achieved power 60%), while males did not exhibit any preference (Z = −0.18, p = 0.9, effect size Cohen’s d = 0.01).

## Discussion

We found that female chicks spontaneously preferred to remain close to a pair of face-to-face hen silhouettes rather than back-to-back. This finding provides evidence that the recognition of face-to-face configuration, likely indicative of interaction, which has been found in infants, adult humans, and adult macaque monkeys, is observed also in birds and does not derive from experience but it is likely to be biologically predisposed in the vertebrate brain. Moreover, given that previous studies in infants never reported a preference for face-to-face stimuli, but only a capacity for discrimination,^18^^,^^20^ the present study is the first report of such an early preference.

While previous studies have demonstrated discrimination and preference for biological motion versus random motion and inverted biological stimuli (reviewed by Vallortigara^17^), our study is also the first to reveal a preference for “face-to-face” interactions between agents over “back-to-back” interactions. Interestingly, this finding shows a different relevant level of information than all previously cited works that investigated spontaneous preferences, e.g. the tendency to approach possible instead of impossible figures,^32^ to prefer consonant music,^33^ or to complete over fragmented objects.^34^ Indeed, in all these cases, chicks were proved to show an innate sensitivity to “natural” configurations in the environment that meet the Gestalt rules of continuity, proximity, and order^35^ as well as the expectation of consonance with the physical rules governing nature. With the present findings, instead, we showed a level of predisposition that reflects expectations about the social world, in opposition to the expectations about the physical world. This could also explain sex differences in this specific predisposition.

Indeed, our experiment revealed a divergence between females and males, with the latter showing no preference. This finding is interesting in itself and aligns with the species’ natural history and the existing ethological literature^28^^,^^29^^,^^30^^,^^31^^,^^36^ (see also for non-human primates Brown et al. study^37^), which indicates that males typically display lower levels of interest in social stimuli and interactions with them compared to females, as part of being a strict polygynous species. Indeed, within adult fowls, there is usually a dominant rooster and many hierarchically organized hens,^38^ favoring the evolution of solitary territorial males and more socially prone females.^39^^,^^40^

Moreover, the two kinds of stimuli used here clearly provided perceptual cues associated with affiliative responses, which are known to be stronger in females. There is evidence that chicks tend to align to the apparent direction of movement of point-light displays.^14^ Thus, facing dyads would elicit approach responses, irrespective of which of the two point-light hens the chick aligns to; instead, non-facing dyads would elicit withdrawal responses.

Additionally, an approach is needed to perform social pecking, which is the main tool for social recognition in young chicks.^41^ Again, social pecking, which is more pronounced in females,^30^ requires proper orienting toward conspecifics, as in facing dyads.

Of course, social interaction is a very general term and may comprise different aspects. For instance, point-light displays facing in the same direction may signal the increased likelihood of a resource between the hens (e.g., corn on the ground between them) that the chicks may be attracted to. This seems unlikely, however, because newly hatched chicks have reserves in the yolk sac for the first two days and are primarily interested in imprinting on a social partner rather than on food at this age.^41^ Besides, there are no obvious reasons why females should be more interested in food than males. It is more likely that chicks may prefer to congregate with groups of hens, which should motivate attraction to two facing hens rather than two hens walking in opposite directions. Whatever the underlying motivation, it is clear that the face-to-face signal to which chicks respond reflects a predisposition in their brain.

### Limitations of the study

The present paper only deals with the issue of the origin (innate or learned) of the face-to-face preference and not with the particular visual cues that generate it (see for specific investigations on this topic Goupil and Papeo works^18^^,^^19^^,^^21^). However, the fact that only females exhibit the preference supports the idea that it is the biological meaning of the face-to-face interaction that elicits interest and not some low-level aspect of the visual stimulation.

## STAR★Methods

### Key resources table

**Table:** 

REAGENT or RESOURCE	SOURCE	IDENTIFIER
**Deposited data**		

Innate sensitivity to face-to-face biological motion dataset	This paper	Figshare: https://figshare.com/articles/dataset/Innate_sensitivity_to_face-to-face_biological_motion_dataset/23713299

**Experimental models: Organisms/strains**		

White Leghorn chicks (*Gallus gallus*)	Azienda Agricola Crescenti, Brescia, IT	Strain: Ross 308

**Software and algorithms**		

DeepLabCut	Mathis et al.^42^	http://www.mackenziemathislab.org/deeplabcut
R studio	Posit	https://posit.co/download/rstudio-desktop/
G-Power 3.1	Faul et al.^43^	https://www.psychologie.hhu.de/arbeitsgruppen/allgemeine-psychologie-und-arbeitspsychologie/gpower
ImprintSchedule	Zanon et al.^44^	https://github.com/MirkoZanon/ImprintSchedule

### Resource availability

#### Lead contact

Further information and requests for resources should be directed to and will be fulfilled by the lead contact, Giorgio Vallortigara (giorgio.vallortigara@unitn.it).

#### Materials availability

This study did not generate new unique reagents.

#### Data and code availability

• Behavioral data have been deposited at Figshare and are publicly available as of the date of publication. DOIs are listed in the key resources table.
• All original code has been deposited at Figshare and is publicly available as of the date of publication. DOIs are listed in the key resources table.
• Any additional information required to reanalyze the data reported in this paper is available from the lead contact upon request.

### Experimental model and study participant details

We studied 240 White Leghorn chicks of the Ross 308 strain (129 females) obtained from a local hatchery (Azienda Agricola Crescenti, Brescia). The estimation was based on an effect of 0.28 (from studies on chicks’ spontaneous preference for motion cues, with similar setup and paradigm; see e.g. Rosa Salva’s work^45^) with the alpha level at 5% and 80% power. The chicks were incubated at the University of Trento under controlled conditions (37.7°C, and 60% humidity). In our pursuit of investigating innate predispositions, we took precautionary measures to ensure that the experimental subjects remained free from external stimuli immediately preceding the experiment. To achieve this, chicks were incubated in the dark in individual small boxes.

A few hours just after hatching (variable between 2 to 10, during which chicks were always kept in the dark), animals were singularly placed in rectangular arenas (60 cm x 90 cm x 90 cm) with high-frequency screens (120 Hz) at opposite ends^44^ to undergo the dual-choice spontaneous task lasting 30 minutes.

The experimental procedures received ethical approval from the University of Trento's Ethical Committee and the Italian Ministry of Health (permit number 324/2022-PR).

### Method details

After hatching, chicks were placed in a rectangular arena (60 cm x 90 cm x 90 cm) with high-frequency screens (120 Hz) at opposite ends.^44^ One screen displayed face-to-face walking point-light silhouette hens, while the other showed a back-to-back configuration (Figure 1A). The stimuli, displayed as GIFs at 13.7 frames per second, were presented during the whole test duration while recording the chicks' behaviors. Previous studies with analogous paradigms focused on the first 6-minute choice; to better evaluate possible adjustments and time variations in the chicks’ spontaneous preference we decided here to record the behaviour for 30 minutes.

### Quantification and statistical analysis

We used DeeplabCut^42^ to track animals’ coordinates, from which we determined the first choice and the time spent near both stimuli. The evaluation was solely based on the animal’s position, considering a stimulus as approached whenever the animal was 30 cm or less from the screen displaying it.

We calculated a preference index for the face-to-face configuration dividing the total time spent close to this stimulus by the total time in proximity of both stimuli.

We used a **χ**^2^ test to examine sex differences in the first choice and a binomial test to assess the preference for the face-to-face configuration. For the analysis in time, a longitudinal non-parametric test was conducted with minutes and sex as factors (using the *nparLD* R package). This is a rank-based test on the marginal distribution functions: using minutes as an ordered factor we can investigate differences in time (null hypothesis: equal marginal cumulative distributions at the different time points). Briefly, the preference index was converted to ranks, data were stratified in independent (i.e., sex) and repeated (i.e., minutes) factors, while mean rank differences between these groups and their interactions were tested using non-parametric ANOVA-type statistics (ATS).

The overall preference (during the cumulative 30 minutes) was evaluated through a permutation ANOVA, and non-parametric Wilcoxon Rank Sum tests were conducted to assess deviations from chance for each sex.

Power analysis and calculation of post-hoc achieved power were performed with G-Power 3.1 software.^43^
